# Commentary: Machine learning in clinical decision-making

**DOI:** 10.3389/fdgth.2023.1214111

**Published:** 2023-07-31

**Authors:** Amanda C. Filiberto, Daniel A. Donoho, Ira L. Leeds, Tyler J. Loftus

**Affiliations:** ^1^ Department of Surgery, University of Florida, Gainesville, FL, United States; ^2^ School of Medicine and Health Sciences, George Washington University, Washington, DC, United States; ^3^ School of Medicine, Yale University, New Haven, CT, United States

**Keywords:** surgery, machine learning, artificial intelligence, decision support, data science

A Commentary on Editorial: Machine learning in clinical decision-making By Filiberto AC, Leeds IL, and Loftus TJ. (2023) Front. Digit. Health. 3:784495. doi: 10.3389/fdgth.2021.784495

Given the success of the Research Topic *Machine Learning in Clinical Decision-Making* published in *Frontiers in Digital Health*, we—the editors of the Research Topic—were pleased to expand the Topic by adding manuscripts that highlight dynamic prediction of mortality among critically ill patients, machine learning models for individualized antimicrobial use duration, electronic health record (EHR) tokenization approaches to patient acuity predictions, and a review of specific artificial intelligence (AI) applications, limitations, and requisites in the United States. These are important Research Topic additions because they embody the principles that clinicians make complex decisions under time constraints and uncertainty using hypothetical-deductive reasoning and individual judgement, which vary from clinician to clinician. Time constraints are imposed by acute diseases and high clinical workloads in which uncertainty results from insufficient knowledge, data, and evidence regarding possible diagnoses and treatments. Clinical decision-support systems often require time-consuming manual data acquisition and entry, which limit their ready adoption by physicians working in high acuity environments with critically ill patients. This General Commentary summarizes key points from the work by Patel, Giordiano, Bolton, Shickel, and their colleagues.

Patients in an intensive care unit (ICU) require close monitoring with a plethora of data points collected in an EHR that are updated frequently. Models predicting mortality have traditionally been used for research purposes rather than individual patient risk assessment at the bedside, and do not consider dynamic clinical status of individual patients. These risk scores are often calculated to produce a score at a single timepoint, overlooking subtle yet important updates in patients' physiology. Despite the rapidly expanding use of EHR data for model training purposes in research environments, current monitoring strategies in clinical use remain limited in their ability to accurately represent changes in patient status.

In volume two of this Research Topic, Patel and colleagues introduce a novel study designed to assess the performance of a dynamic method of updating mortality risk every three hours using a criticality index mortality (CI-M) neural network methodology (1). The data were collected from 2018 to 2020 at the Children's National Hospital, comprising 72 pediatric ICU beds. EHR data were extracted and ICU courses were stratified into three-hour intervals, using a neural network to predict outcomes. The CI-M uses a neural network which incorporates physiology, therapy, and intensity of care to compute a morality risk for pediatric ICU patients in a clinically relevant model using updated data every three hours. The area under the receiver operating characteristic curves had a minimum value of 0.778 (95% confidence interval 0.689–0.867) at hour three and a maximum value of 0.885 (0.841,0.862) at hour 81. The ten most important variables for risk prediction were duration of ICU stay, ventilator-free days, hours on mechanical ventilation, coma scores, age, and neutrophil counts. The CI-M has the potential to enhance prognostic assessments of critically ill pediatric patients, toward improving clinical decision-making and care. Ideally, this risk model will be externally validated and applicable to other institutions.

Bacterial antimicrobial resistance is a global threat and is associated with increased risk of mortality not only for index patients who develop resistant infections, but also for other patients who suffer collateral harm from spread of resistant organisms, often through healthcare worker vectors. Clinical decision support systems have the potential to increase antimicrobial stewardship, thus mitigating antimicrobial resistance. Bolton et al. use a machine learning and synthetic control-based approach to estimate patients’ length of stay (LOS) and mortality outcomes for any given day if they were to stop vs. continue antibiotic treatment (2). Comparisons between decision support system use and control experiences demonstrated minimal difference for both stopping and continuing scenarios, indicating that decision support estimations were reliable (average LOS differences of 0.24 and 0.42 days, respectively). Their approach is novel, can assist with individualized antibiotic cessation, and establishes the safety of patient-specific shortening of antibiotic treatment durations.

Shickel et al. describe their use of a transformer-based patient acuity prediction framework in the critical care setting with a data embedding scheme that captures both concept and corresponding measurement values of many disjoint clinical descriptors (3). The authors introduce a mechanism for combining both absolute and relative temporality as an improvement over traditional positional encoding. They highlight the future of this promising approach while noting that more research is needed to emphasize analyzing self-attention distributions between input variables and clinical outcomes to further the clinical understanding and enhance the trust of clinicians using transformers in healthcare settings.

In a comprehensive review of peer-reviewed literature describing access to AI for clinical decision making, Giordano et al. highlight the use of machine learning models for risk stratification, early warning of acute decompensation, potential bias in machine learning algorithms, and the paradigm shift in medical training towards emerging biomedical informatics applications (4). With the widespread adoption of EHRs there are vast repositories of data sets that are ideal for AI training and testing, and many healthcare disciplines have developed and validated promising solutions for improved risk stratification and optimization of patient outcomes. Healthcare workers will be expected to comfortably work within this new AI frontier and, in turn, relate it to their patients. This review provides an optimal overview and introduction to the novel methods that should be considered.

We hope that you have enjoyed and learned from these important additions to the *Machine Learning in Clinical Decision-Making* and *Machine Learning in Clinical Decision-Making—Volume II* Research Topics published in *Frontiers in Digital Health*.

## References

[1] Patel Anita K Trujillo-Rivera E Morizono H Pollack MM. The criticality Index-mortality: a dynamic machine learning prediction algorithm for mortality prediction in children cared for in an ICU. Front Pediatr. (2022) 10:1023539. 10.3389/fped.2022.1023539 36533242 PMC9752098

[2] Bolton WJ Rawson TM Hernandez B Wilson R Antcliffe D Georgiou P Machine learning and synthetic outcome estimation for individualised antimicrobial cessation. Front Digit Health. (2022) 4:997219. 10.3389/fdgth.2022.997219 36479189 PMC9719971

[3] Shickel B Silva B Ozrazgat-Baslanti T Ren Y Khezeli K Guan Z Multi-dimensional patient acuity estimation with longitudinal EHR tokenization and flexible transformer networks. Front Digit Health. (2022) 4:1029191. 10.3389/fdgth.2022.1029191 36440460 PMC9682245

[4] Giordano C Brennan M Mohamed B Rashidi P Modave F Tighe P. Accessing artificial intelligence for clinical decision-making. Front Digit Health. (2021) 3:645232. 10.3389/fdgth.2021.645232 34713115 PMC8521931

